# Twist reverses muscle cell differentiation through transcriptional down-regulation of myogenin

**DOI:** 10.1042/BSR20130068

**Published:** 2013-12-03

**Authors:** Nikolaos P. Mastroyiannopoulos, Antonis A. Antoniou, Andrie Koutsoulidou, James B. Uney, Leonidas A. Phylactou

**Affiliations:** *Department of Molecular Genetics, Function and Therapy, The Cyprus Institute of Neurology and Genetics, Nicosia, Cyprus; †The Henry Wellcome Laboratories for Integrative Neuroscience and Endocrinology, University of Bristol, Bristol, U.K.

**Keywords:** dedifferentiation, myogenesis, myogenin, myotubes, Twist, AdC, control adenoviral vector, AdMyoD, MyoD-overexpressing adenoviral vector, AdT, TWIST-overexpressing adenoviral vector, Ara-C, cytosine β-D-arabinofuranoside, bHLH, basic helix-loop-helix, ChIP, chromatin immunoprecipitation, DM, differentiation medium, EdU, 5-ethynyl-2′-deoxyuridine, GAPDH, glyceraldehyde-3-phosphate dehydrogenase, GM, growth medium, MEF, myocyte enhancer factor, MRF, myogenic regulatory factor

## Abstract

Some higher vertebrates can display unique muscle regenerative abilities through dedifferentiation. Research evidence suggests that induced dedifferentiation can be achieved in mammalian cells. TWIST is a bHLH (basic helix-loop-helix) transcription factor that is expressed during embryonic development and plays critical roles in diverse developmental systems including myogenesis. Several experiments demonstrated its role in inhibition of muscle cell differentiation. We have previously shown that overexpression of TWIST can reverse muscle cell differentiation in the presence of growth factors. Here we show that TWIST reverses muscle cell differentiation through binding and down-regulation of myogenin. Moreover, it can reverse cellular morphology in the absence of growth factors.

## INTRODUCTION

The course of myogenesis is a well-characterized example of terminal differentiation. Myoblasts are capable of proliferation and upon demand to form skeletal muscle, these cells exit the cell cycle and through the activation of muscle-specific transcription factors they fuse into multinucleated terminally differentiated myotubes [1,2]. MRFs (myogenic regulatory factors), myogenin, MyoD, MRF4 (Myf6) and Myf5 are bHLH (basic helix-loop-helix) transcription factors that regulate myogenesis [3–9]. Myogenin is essential during differentiation. Mice lacking the *myogenin* gene die at birth due to severe skeletal muscle deficiency, as myoblasts are unable to fuse into multinucleated myotubes [10]. Furthermore, MyoD and Myf5 are unable to substitute myogenin's function during differentiation [11]. Moreover, mice lacking the *myogenin* gene express normal levels of MyoD and Myf5 [10].

Unlike mammals, vertebrates such as zebrafish and salamanders can display unique regenerative abilities through dedifferentiation or differentiation of precursor cells [12]. Following injury, these vertebrates are able to induce reversal of the differentiation state, which leads to a series of events that aim to generate proliferating regenerative progenitor cells with the ability to restore the lost tissue in a precise way [12–14]. Some research groups have attempted to induce dedifferentiation of muscle cells by exogenous genes or chemicals. Mouse C2C12 myotubes treated with limb regeneration extracts were able to induce myotubes to reenter the cell cycle, exhibited reduced levels of muscle differentiation proteins and cleaved to produce smaller myotubes or proliferating mononucleated cells [15]. In another study, combination of growth medium and ectopic msx1 expression caused the reduction of muscle-specific proteins and the cleavage of these myotubes into proliferating mononucleated cells that were able to redifferentiate into muscle or trans-differentiate into various cell types [16]. Microinjection of Barx2 cDNA into immature myotubes derived from primary cells led to cleavage and formation of mononucleated cells that were able to proliferate [17]. Using a chemical approach, terminal differentiated myotubes were incubated with a triazine compound. Myotubes showed to cellularize into smaller myotubes or mononucleated cells, which were able to survive and divide [18]. Similarly, myoseverin, a trisubstituted purine, was shown to induce reversible fission of multinucleated myotubes into mononucleated cells, which were able to enter the cell cycle [19]. Recently, mammalian skeletal muscle cells were induced to dedifferentiate into proliferating mononuclear cells following treatment with myoseverin and temporary p21 suppression. These cells were further induced to act as multipotent stromal cells by further treatment with the small molecule, reversine (2-(4-morpholinoanilino)-6-cyclohexylaminopurine) and simple chemical modifications of the culture media [20]. When cell cycle inhibitors, p21 and p27 were depleted from terminal differentiated mouse myotubes, incomplete DNA replication and apoptosis was observed. In contrast, when p21 and p27 were depleted from quiescent, non-terminal differentiated fibroblasts and muscle cells, DNA replication was fully recovered and apoptosis was no longer observed. These cells were able to proliferate in the absence of growth factors [21]. Recently, evidence for natural dedifferentiation of muscle cells following injury was reported by using a Cre/Lox-β-galactosidase system [22,23]. Finally, we have recently reported that down-regulation of myogenin leads myotubes to a reversal of muscle cell differentiation [24].

TWIST is a bHLH transcription factor initially identified in *Drosophila* [25]. *Twist* orthologues have subsequently been identified in other species, including mouse and human [26,27]. It forms functional homodimers as well as heterodimers with various bHLH protein partners and binds to the promoter of target genes. TWIST is expressed during embryonic development and plays critical roles in diverse developmental systems such as mesoderm formation, myogenesis, cardiogenesis and neurogenesis [28].

Several experiments, mainly involving overexpression of TWIST in cell lines, have demonstrated its role in inhibition of muscle cell differentiation. Nevertheless, in *Drosophila*, TWIST has been reported to promote myogenesis [29], whereas in mice it inhibits muscle cell differentiation [30,31]. Published results, mainly by overexpression of TWIST in cell lines and mice, show that TWIST inhibits myogenesis by inhibiting MyoD, which is activated by binding to MEF (myocyte enhancer factor)-2 and E-proteins [25,32]. Not a single mechanism has been assigned to MyoD inhibition by TWIST. There is however evidence of direct TWIST binding to MyoD [33] or TWIST binding and sequestration of MEF-2 or E-proteins [34,35].

We have previously shown that overexpression of TWIST in terminally differentiated myotubes caused their cleavage to mononucleated cells in the presence of growth factors and re-entry to the cell cycle. This was accompanied by a reduction of MRF (myogenic regulatory factor) levels [36]. Here, we wanted to investigate the direct mechanism by which TWIST reverses muscle cell differentiation. We show that TWIST causes reversal of muscle cell differentiation through binding and down-regulation of myogenin. Moreover, reversal of cellular morphology (myotube fragmentation) was possible through this pathway, in the absence of growth factors.

## MATERIALS AND METHODS

### Tissue culture

C2C12 mouse myoblasts (ECACC) were grown to confluency under 5% (v/v) CO_2_ at 37°C in the GM (growth medium), DMEM (Dulbecco's modified Eagle's medium) (Gibco) supplemented with 10% (v/v) FBS (Gibco), 2 mM glutamine (Gibco) and penicillin-streptomycin (100 μg/ml–100 units/ml) (Gibco). For C2C12 muscle cell differentiation, cells were then switched to DM (differentiation medium), DMEM supplemented with 2% (v/v) horse serum (Gibco), 2 mM glutamine and penicillin–streptomycin (100 μg/ml–100 units/ml) for 4 days. During the first 2 days of differentiation, Ara-C (cytosine β-D-arabinofuranoside) (Sigma) (4 μg/ml) was included in order to eliminate most undifferentiated myoblasts (for time-lapse microscopy purposes). Medium was then replaced with the fresh DM medium without Ara-C in the presence or absence of adenoviral vectors, for another 2 days (day 4 of differentiation). For adenoviral transductions on differentiated myotubes, 100 MOI (multiplicity of infection) of each of the adenoviral vectors (VectorBiolabs) was used. Cells were maintained in 37°C/5%CO_2_ or in a temperature and CO_2_-regulated time-lapse microscope (AxioVision, Zeiss).

### Chromatin immunoprecipitation

C2C12 cells were grown to confluency after which DM was added. On day 2 of differentiation cells were transfected with AdT (TWIST-overexpressing adenoviral vector) or AdMyoD (MyoD-overexpressing adenoviral vector). ChIP (chromatin immunoprecipitation) assay was performed on 4-day differentiated C2C12 myotubes using MAGnify™ Chromatin Immunoprecipitation System (Invitrogen) according to the manufacturer's instructions. Briefly, dynabeads were coupled to the MyoD and TWIST primary antibodies (Abcam) prior to the crosslinking of chromatin. Following cell lysis, samples were subjected to chromatin binding to the antibody dynabeads complexes in order to isolate only the DNA of interest after a series of washes. The cells were immunoprecipitated using TWIST or MyoD antibodies, respectively (Abcam). For ChIP assay, two controls were used: a positive and a negative control antibody. For positive control, 2.5 μg of unconjugated polyclonal antibody specific to human and mouse histone H3, trimethylated at lysine 9 [K9me3] (H3-K9Me3) (Invitrogen) was added. For negative control, 1 μg of rabbit or mouse IgG antibody was used.

Primers for ChiP assay were E1 F: 5′- GTTTCTGTGGCGTTGGCTAT -3′, E1 R: 5′ AAGGCTTGTTCCTGCCACT 3′. E2 F: 5′- AATCAAATTACAGCCGACGG -3′, E2 R: 5′- GAAACGTCTTGATGTGCAGC -3′. E3 F: 5′- CAAGGAACTGAAGGGGTCTG -3′, E3 R: 5′- CCCTGTACTGGGGCATATAGTT -3′. Sat2 F: 5′- AGCAGATGGCTTTGGAGAGA 3′, Sat2 R: 5′- CTGGGAGCAACCCTTATTCA -3′ (Table 1).

**Table 1:** PCR conditions for ChIP assays

DNA fragment	Enzyme	PCR no. cycles	PCR annealing temp.	Expected fragment size
E1	Taq polymerase (Qiagen)	27	57°C	122 bp
E2	Taq polymerase (Qiagen)	27	56°C	140 bp
E3	Taq polymerase (Qiagen)	25	55°C	168 bp
Sat2	Taq polymerase (Qiagen)	25	58°C	247 bp

### Luciferase assays

**Figure 1:**
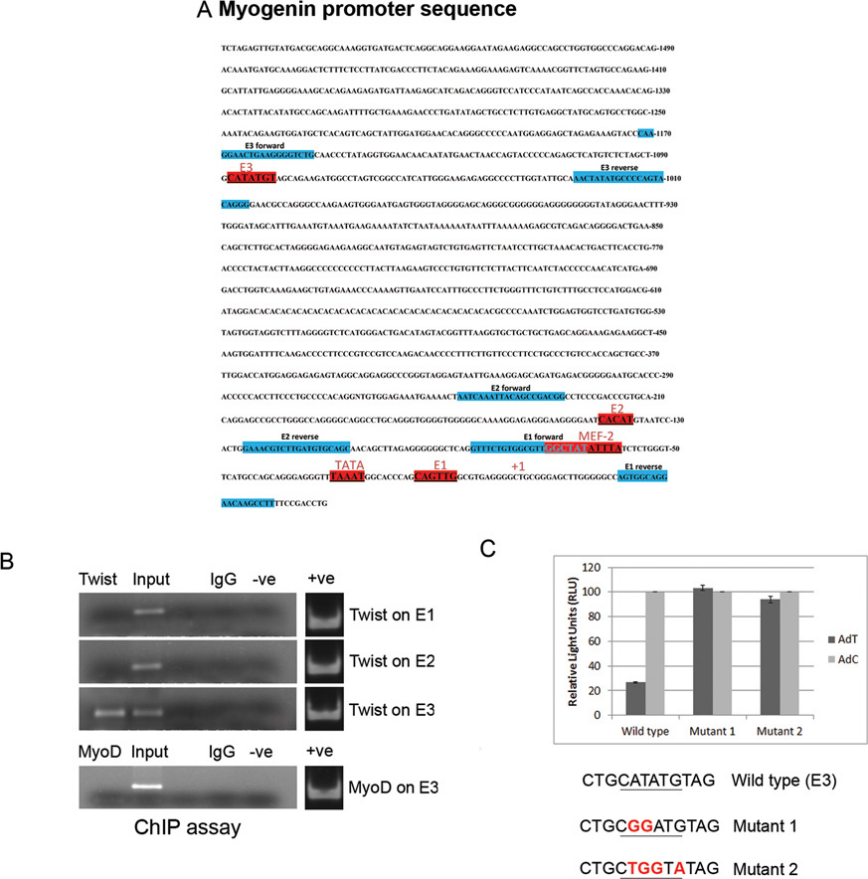
TWIST binds to *myogenin* promoter and down-regulates its gene expression (**A**) *Myogenin* promoter sequence containing the two classical conserved E boxes (E1 and E2) within 143 bp of the transcription start site and a third non-conserved E box (E3), 1083 bp upstream of the transcriptional start site. E boxes are shown in red. Forward and reverse primers designed to amplify the E1, E2 and E3 boxes are highlighted in blue. (**B**) ChIP assay revealed that following TWIST overexpression, TWIST bound to the E3 box. On the other hand, TWIST did not bind to E1 and E2 boxes. Similarly, following MyoD overexpression, ChIP assay specific for the E3 box showed no binding of MyoD. (**C**) Luciferase assay showed that binding of TWIST (AdT) to *myogenin* promoter resulted in down-regulation of gene expression, compared with control-transduced cells (AdC), which was abolished by the introduction of specific mutations in the bound E-box sequence (Mutants 1 and 2).

Mouse *myogenin* promoter, as shown in figure 1, was cloned into a luciferase-pcDNA3 plasmid upstream of the luciferase gene. The *myogenin* promoter/luciferase plasmid was mutated using the GeneArt Site-Directed Mutagenesis System (Invitrogen) at *myogenin* promoter E-box site (CATATG) (E3) by using two different sets of primers: Mutant 1 F: 5′- AGAGCTCATGTCTCTAGCTGCGGATGTAGCAGAA -3′, R: 5′- GCAGCTAGAGACATGAGCTCTGGGGGTACTGG -3′ and Mutant2 F: 5′- AGAGCTCATGTCTCTAGCTGCTGGTATAGCAGAAGAT -3′, R: 5′- GCAGCTAGAGACATGAGCTCTGGGGGTACTGG -3′. C2C12 cells were stably transfected with the wild-type and mutant *myogenin*/luciferase plasmids. Cells expressing the wild-type or mutant *myogenin* promoters were differentiated for 2 days before being transfected with AdT or AdC (control adenoviral vector) after which cells were differentiated for a further 2 days. Luciferase gene expression was then measured using Dual-Luciferase Reporter Assay (Promega). Cells were lysed and mixed with Luciferase Assay Substrate (LAR II) before measuring activity in a luminometer (Berthold). In order to ensure that luciferase readings reflect results originating from viable cells, the viability of cells in experiment was measured using a cell counter (Countess Automated cell counter, Invitrogen).

### Cell cycle studies

Cell cycle studies were performed using Click-iT EdU Imaging kit (Invitrogen). Myotubes were transfected with adenoviral vectors (day 2 of differentiation) and incubated in GM or DM for a further 2 days. Cells were then assayed with EdU (5-ethynyl-2′-deoxyuridine) for 3 h (Invitrogen). Following incubation with EdU cells were then fixed with 4% (v/v) paraformaldehyde in PBS for 20 min, washed twice with 3% (w/v) BSA in PBS, before and after permeabilization with 0.5% (v/v) Triton-X-100 in PBS for 20 min at room temperature (25°C) prior to 30 min incubation in a mixture containing Alexa fluor 647. Nuclear staining was performed with DAPI II (1.5 ng/μl) (Vysis) and observed under a fluorescence microscope (AxioVision, Zeiss).

### RNA analysis

Total RNA was extracted from transfected or untransfected myotubes (Perfect RNAEukaryotic Mini kit, Eppendorf) and 500 ng of RNA was subjected to reverse transcription using MuLV reverse transcriptase (NEB). For PCR, mouse-specific primers were used for the analysis of expression for the following molecules: MyoD F 5′- AGTGAATGA GGCCTTCGAG A -3′, R 5′- GCATCTGAGTCGCCACTGTA -3′. Myogenin F 5′-CATCCAGTACATTG AGCGCCTA-3′, R 5′- GAGCAAATGATCTCCTGGGTTG -3′. Myf6 F 5′- ATG GTACCCTATCCCGTTGC -3′, R 5′- TAGCTGCTTTCCGACGATCT -3′. Myf5 F 5′- TGAAGGATGGACATGACGGACG -3′,R 5′-TTGTGTGCTCCGAAGGCTGCTA -3′. Cyclin D1 F 5′- GGCACCTGGATTGTTCTGCT -3′, R 5′- CAGCTTGC TAGGGAACTTGG -3′. Cyclin E2 F 5′ -GGAACCACAGATGAGGTC -3′, R 5′- CG TAAGCAAACTCTTGGAG -3′. Pax7 F: 5′- GAGTTCGATTAGCCGAGTGC -3′, Pax7 R: 5′- CGGGTTCTGATTCCACATCT -3′. GAPDH F 5′-TCATCATCTCCGCCC-CTTCT-3′, R 5′- GAGGGGCCATCCACAGTCTT -3′ (Table 2).

**Table 2:** PCR conditions for RNA analysis. Experiments were repeated at least three times and gel bands were measured using the Image J software

DNA fragment	Enzyme	PCR no. cycles	PCR annealing temp.	Expected fragment size
MyoD	Taq polymerase (Qiagen)	27	57	222 bp
Myog	Taq polymerase (Qiagen)	27	59	181 bp
Myf6	Taq polymerase (Qiagen)	25	57	183 bp
Myf5	Taq polymerase (Qiagen)	24	62	134 bp
Cyclin D1	Taq polymerase (Qiagen)	27	59	232 bp
Cyclin E2	Taq polymerase (Qiagen)	26	58	227 bp
GAPDH	Taq polymerase (Qiagen)	23	61	220 bp
Pax7	Taq polymerase (Qiagen)	30	56	170 bp

### Western blot

Cells were lysed using a protein lysis buffer including protease inhibitor. 40–60 μg of protein extracts were incubated with myogenin (1/200; BD), MyoD, Myf5, Myf6 (1/300; Santa-Cruz), cyclin D1 (1/400; Abcam), cyclin E2 (1/200, Abcam) and GAPDH (glyceraldehyde-3-phosphate dehydrogenase) (1/2000; Santa-Cruz) primary antibodies followed by incubation with goat anti-mouse IgG or donkey anti-rabbit IgG secondary antibodies conjugated to horseradish peroxidase (Santa-Cruz).

## RESULTS

### TWIST binds *myogenin* promoter and down-regulates its expression

We have recently shown that down-regulation of myogenin can reverse muscle cell differentiation by cleavage into mononuclear cells which enter the cell cycle. Since TWIST is known as a transcription factor that negatively regulates muscle cell differentiation, we first looked at the putative E-boxes on the mouse *myogenin* promoter, which is located on chromosome 1 and is 1.5 kb in length (chr1:134288446–134290053) (Figure 1A). TWIST is known to bind to E-boxes. With this in mind, we tested the binding affinity of TWIST with E-boxes found in *myogenin* promoter. In order to determine whether Twist can bind to the *myogenin* promoter and therefore contribute to the mechanism of reversal of differentiation, C2C12 muscle cells were first differentiated *in vitro* to multinucleated myotubes and then transduced with an AdT. Following incubation with the viral vector, the ability of TWIST to bind to the *myogenin* promoter was tested by ChIP. As it can be seen in Figure 1B, TWIST was able to bind the *myogenin* promoter. This was possible only for an E-box (E3) that was found 1083 bp upstream of the transcription start site. The two well-characterized E-boxes (E1 and E2) of the *myogenin* promoter showed not to have binding affinity with TWIST (Figure 1B). Furthermore, a AdMyoD was *in vitro* transduced in differentiated myotubes using the same conditions to the previous AdT transfections. This was done in order to investigate whether MyoD interacts with the same E box (E3) and acts in competition to TWIST protein. Following adenoviral transductions of MyoD, ChIP assay showed that MyoD did not interact with E3 (Figure 1B). In order to determine the effect of the binding of TWIST to *myogenin*, *myogenin* promoter (sequence shown in Figure 1A) was cloned upstream of the *luciferase* gene in a plasmid and a stable cell line was created. Following transduction of AdT, luciferase activity was considerably reduced, whereas no reduction was observed in the transduction with a control AdC (adenoviral vector) (Figure 1C). This result demonstrates that TWIST is able to bind and down-regulate *myogenin* promoter activity. As a next step, two specific mutations (mutants 1 and 2) were introduced into the E3 site in order to determine the specificity of binding. In both mutants, the activity of luciferase remained unchanged, similar to AdC denoting that TWIST binding was specific.

### TWIST-mediated down-regulation of myogenin induces cellularization in the absence of serum

Based on our previous reports, myogenin down-regulation caused reversal of muscle cell differentiation with cellular and molecular characteristics which resemble those of TWIST-mediated reversal of differentiation [24,36]. Binding of TWIST to *myogenin* promoter indicates that TWIST mediates its effect via myogenin down-regulation. All previous reports investigating induced reversal of differentiation included the addition of growth factors in the procedures. Growth factors are essential for the proliferation of muscle cells since they induce pathways involved in cell division. *In vitro* differentiation, on the other hand proceeds in the absence of growth factors since cells must exit the cell cycle. Previous experiments have shown that newt myotubes are able to dedifferentiate in the absence of growth factors [37]. The possibility that TWIST induces reversal of muscle cell differentiation in the absence of growth factors was next investigated.

**Figure 2:**
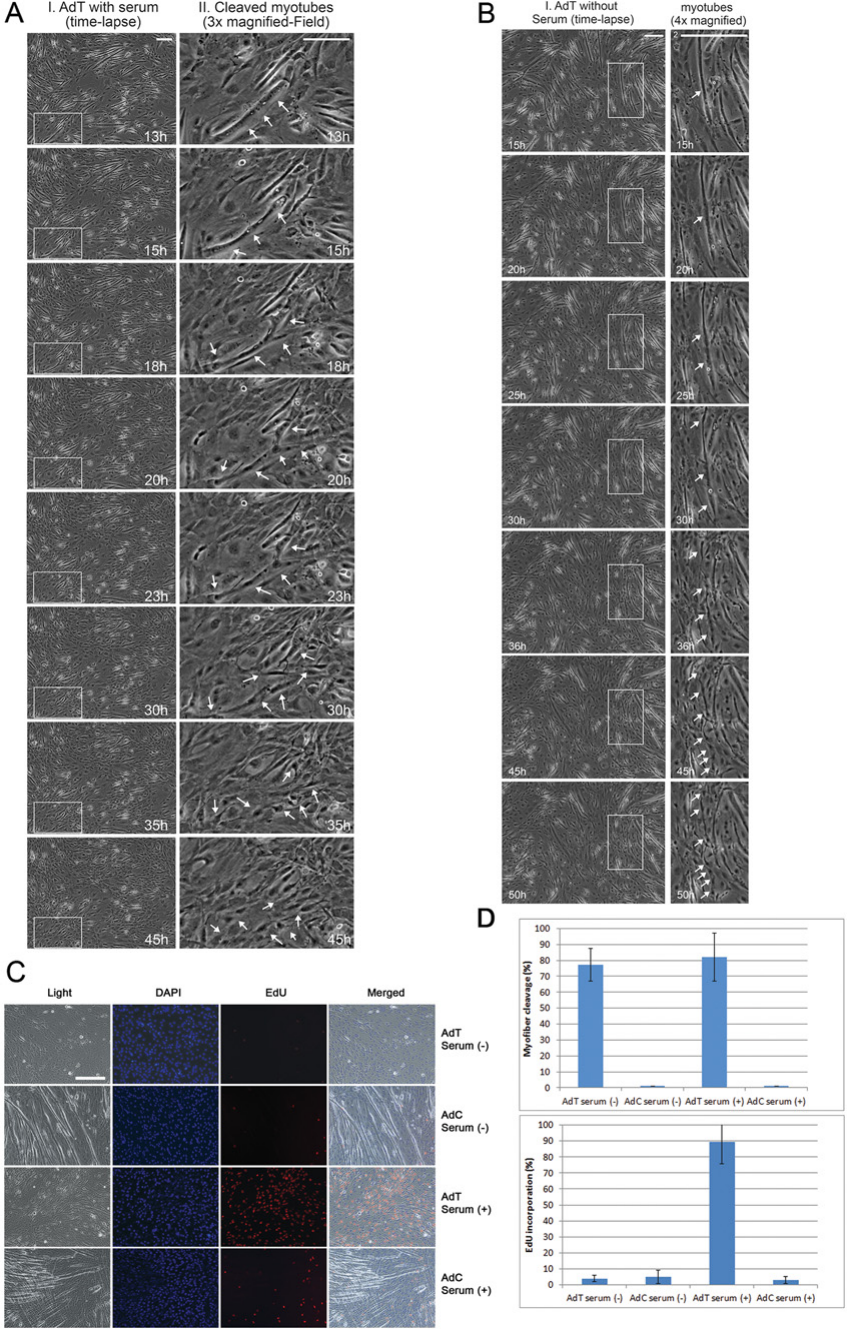
TWIST-mediated muscle cell morphological reversal occurs in the absence of growth factors (**A**) Following myotube differentiation, cells were transfected with AdT. GM was then added to the cells and placed for time lapse microscopy. (I) Myotubes after AdT transfection (scale, 250 mm). (II) Myotube in higher magnification from 13 to 45 h displayed significant morphological changes and cleavage. During 18–30 h, myotubes began to morphologically change (arrows indicate the movement of the nuclei and the areas of possible cleavage). During 30–45 h, myotubes were completely cleaved into mononucleated cells (arrows indicate cleaved cells). (**B**) Following myotube differentiation, cells were transfected with AdT. Fresh DM medium was then added to the cells and placed for time-lapse microscopy. (I) Myotubes after AdT transfection (scale, 250 mm). (II) Myotubes in higher magnification from 15 to 52 h displayed significant morphological changes and cleavage. During 24–38 h, myotubes began to morphologically change (arrows indicate the movement of the nuclei and the areas of possible cleavage). During 38–52 h, myotubes were completely cleaved into mononucleated cells (arrows indicate cleaved cells). (**C**) Following myotube differentiation, cells were transfected with AdT and AdC in the presence or absence of growth factors. Cell cycle activity was assayed by EdU. Cells transfected with AdT in the absence of growth factors showed significant cellularization with minimal EdU activity. Cells transfected with AdT in the presence of growth factors showed significant cellularization and high EdU activity. No cellularization effect was observed when cells transfected with AdC in the presence or absence of growth factors. (D) Quantification of cleaved myotubes and EdU incorporation after AdT transfections with or without growth factors compared with AdC transfected cells. Results were obtained by measuring myofibres and EdU-positive cells in three random areas using ×10 magnifications.

Differentiated muscle cells were transduced with AdT and then induced to reverse their cellular fate and morphology by the addition or the absence of growth factors. Following cell incubation under a time-lapse microscope, around 80% of myotubes were shown to be cleaved in individual mononuclear cells with and without growth factors, compared with control-transduced cells (Figures 2A, 2B and 2D). To determine whether product cells initiated DNA synthesis EdU incorporation assays were performed on product cells that originated from the cleavage of myotubes in the absence of growth factors, did not show any EdU incorporation. In contrast, DNA synthesis was seen in approximately 90% of product cells in the presence of growth factors (Figures 2C and 2D). In conclusion, although cells were morphologically changed, indicating reversal of differentiation, the absence of growth factors prevented them from entering the cell cycle. This result indicates that although TWIST cannot sustain a full reversal of differentiation (cell cycle re-entry) in the absence of growth factors, it is capable of reversing the morphology of differentiated muscle cells.

**Figure 3:**
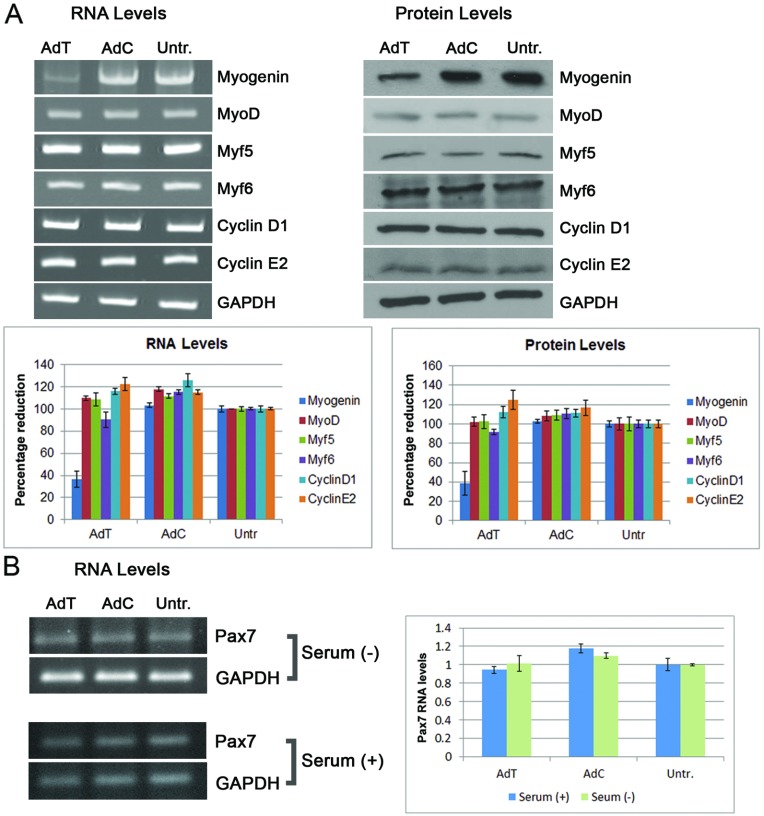
TWIST-mediated muscle cell morphological reversal in the absence of growth factors is driven by down-regulation of myogenin (**A**) Overexpression of TWIST (AdT) in the absence of growth factors reduced myogenin RNA and protein levels but did not affect the levels of MyoD, Myf6, Myf5, CyclinD1, CyclinE2 compared with control-transduced cells (AdC) or untransduced cells (Untr.). GAPDH served as an internal control. (**B**) Overexpression of TWIST (AdT) in the presence or absence of growth factors had no effect on Pax7 expression as compared with control-transduced cells (AdC) or untransduced cells (Untr.). Images were analysed and quantified by Image J software. Data represent the mean+S.D. from at least three independent experiments in triplicate.

In order to investigate the molecular changes of the absence of growth factors in the reversal of muscle cell differentiation, RNA and protein analysis was performed in TWIST-transduced and control myotubes. Our previous work demonstrated that the presence of growth factors in the AdT transductions induced the expression of cell cycle molecules such as cyclin D1 and suppressed the MRFs. Here, as it can be seen in Figures 3(A) and 3(B), the absence of growth factors had no influence on any of the cell cycle molecules or MRFs. The only molecule that is altered is myogenin which was found reduced compared to control-transduced cells. This result indicates that in the absence of growth factors, TWIST specifically binds the *myogenin* promoter, resulting in the down-regulation of myogenin, which drives the reversal of myotube morphology causing the cleavage of myotubes into individual mononuclear cells. Finally, the expression levels of Pax7 were assessed, since this molecule is known to be reduced when myogenin is increased [38]. Following experimentation, PAX7 was found unchanged following TWIST overexpression, both in the presence and absence of growth factors (Figure 3B).

## DISCUSSION

This study reports a novel pathway which leads to the reversal of muscle cell differentiation. Moreover, results from this study indicate that TWIST can achieve reversal of muscle cell differentiation (cleavage of myotubes into mononuclear cells) in the absence of growth factors.

As of the date of submission, there is no strong evidence indicating that dedifferentiation of muscle cells occurs in mammals. There is however published work which shows that following induction with genetic or chemical molecules, reversal of differentiation is plausible. Since this pathway occurs naturally in some higher vertebrates as an additional way for regenerating muscle, it may well be beneficial for humans, if appropriate inducers can mimic this phenomenon. Induced reversal of muscle differentiation may also reveal unknown functions of molecules and novel pathways in the regulation of myogenesis. For example, reversal of muscle differentiation by down-regulating myogenin shows the important role of this MRF to maintain differentiation [24]. Interestingly, from previous studies Pax7 was found to be down-regulated as myogenin was induced. In our case however, Pax7 was not found altered following down-regulation of myogenin by TWIST either in presence or absence of growth factors [38]. This could imply that the molecular mechanism of dedifferentiation following overexpression of TWIST may involve such pathways which do not lower the levels of Pax7.

Results from this study reveal a mechanism by which overexpression of TWIST reverses muscle cell differentiation. Binding of TWIST to the E-box of the promoter of *myogenin* suppressed its activity and down-regulated its expression. As a result, differentiation is reversed by the subsequent reduction in the other MRFs and the entry of the product cells to the cell cycle. Interestingly, MyoD was not found to bind to the same E box that TWIST binds, implying that there is no competition in the particular E box, although MyoD has been previously shown to bind to the *myogenin* promoter [39,40]. Findings from this work show also that TWIST-mediated myogenin reduction leads, independently of cell cycle entry, to the cell morphological reversal of differentiation, i.e. the myotube cleavage into mononucleated cells. This, not only demonstrates that TWIST is a potent inducer of morphological reversal of muscle cells but it also indicates that myogenin down-regulation is responsible for the cleavage of terminally differentiated myotubes into mononucleated cells independently from the cell cycle re-entry.

This work provides mechanistic insights to TWIST-mediated reversal of muscle cell differentiation, which might be beneficial for muscle regeneration strategies and also for the investigation of novel pathways in myogenesis.
